# Dental Hygiene

**Published:** 1897-07

**Authors:** J. Clifford Wing


ARTICLE IV.

### DENTAL HYGIENE.

BY J. CLIFFORD WING.

One of the chief duties of the dentist is to remedy the ravages of dental caries and to use all possible means of averting it. "Prevention is better than cure," and this is especially so with the teeth. Various digestive changes are constantly going on in the mouth. Starch is being converted into grape sugar, and lactic and other acid fermentations are always taking place. The particles of food which lodge in the interstices of the teeth form a pabulum for the multiplication and habituation of micro-organisms, which turn the food into a fermenting mass, and this, being of an acid reaction, is highly deleterous to the tooth structure. This is especially so in dirty mouths, in which fermentation and putrefaction are allowed to continue undisturbed.

How are we to combat these forces? It is needful that the tooth germ should have the proper constituents supplied to it for its growth and calcification, both before and after birth of the child. Therefore the diet of the mother should, as far as is practicable, include such elements as are fitted for the nourishment of these organs, with what is required for the general support of the child. Whole-meal bread is preferable to ordinary white bread, from which all the tooth and bone forming constituents of the grain have been eliminated. It is chiefly the outer portions of the grain which contain the tooth-forming phosphates, and these are generally discarded in the manufacture of flour, so as to produce a whitier article. That which is discarded is full of nutriment for the teeth; that which is retained is little more nutritious than starch. Oatmeal, and like substances, are valuable articles of diet for tooth and bone-forming purposes.

Then, after birth, the food should be of the necessary character to produce well calcified teeth, for the few short years of childhood settle the quality of the teeth of a lifetime.

The best diet for the infant is the natural milk, and later cow's milk. Milk has been said to be "the food prepared by Nature for the maintenance of the young," and as it contains most of the substances needed in the formation of the teeth, as well as those for the general welfare of the child, it should be the staple article of diet during infancy, and its use should be continued throughout childhood and youth. When the child is old enough, the use of oatmeal and whole-meal bread, as a part of the daily meal, will tend to produce teeth good in structure and well calcified, and therefore resistant of decay. As the dentine and tooth-pulp always exert vital action against caries it will be seen that diet is of importance throughout life to the welfare of the teeth.

The most essential agent in the preservation of the teeth is cleanliness, for so great are the powers of the decay that unless kept clean and aseptic the best of teeth will be likely to fall a prey to caries. To a pleasing and and agreeable expression of the face a clean and healthy denture is of the greatest consequence. We all know the difference between a clean and unclean mouth. The clean mouth, with its bright looking teeth free from deposit, will look pleasant, even though the teeth themselves may not be regular. The gums will be healthy, and the breath pure and sweet. The unclean mouth, on the other hand, contains dull, dirty teeth, covered at the margins with deposit, the gums is reddened at the edges, and soft and spongy, and the breath—well, most of us can speak from experience on that subject after our extraction mornings, and the less said the better. So that both for the preservation and appearance of the teeth it is of the greatest importance to pay attention to cleanliness. A person can not be brought too early into habits of strict cleanliness; a tooth-brush can scarcely be placed too early into the child's hands with instruction how to use it.

The tooth-brush must be fairly small, with bristles not too closely set together, and of medium stiffness. It is preferable to have the brush notched crosswise, and to have the central rows of bristles longer than the outer. It should always be kept in the open air, and certainly not shut up in a closed vessel. It is useful to have an alternation of two or more brushes in use at a time. It is hardly necessary for me to say that the teeth should be cleansed night and morning, especially at night, for if not, the particles of food are left undisturbed for some hours to ferment and putrefy and exert their deleterious action on the teeth; whereas, if the brush has been used, all these agents of decay have been removed. The practice of giving children a biscuit or something sweet on going to bed must be strongly condemned, as it fills the interstices of the teeth with highly fermentable material, which remains to continue its injurious action while the child sleeps.

To return to the use of the tooth-brush—the movements should not so much be the usual horizontal action, but an up and down movement, sweeping the *debris* from the gum margin to the crown. In the former movement, the spaces between the teeth are hardly touched, indeed, *debris* is often swept into them, while in the latter the interstices and all parts of the teeth are cleansed.

Patients should be made awars of the fact that they are, or ought to be, cleaning the teeth, not for appearance so much as for preservation, and that it is essential to brush the back teeth as well as the front and all surfaces. In fact the teeth should be brushed in all those parts which are accessible to the tooth brush.—*British Journal.*

